# Identification and functional analysis of differentially expressed genes in poorly differentiated hepatocellular carcinoma using RNA-seq

**DOI:** 10.18632/oncotarget.16415

**Published:** 2017-03-21

**Authors:** Yi Huang, Jianbo Pan, Dunyan Chen, Jiaying Zheng, Funan Qiu, Feng Li, Yanan Wu, Wenbing Wu, Xiaoli Huang, Jiang Qian

**Affiliations:** ^1^ Department of Clinical Laboratory, Fujian Provincial Hospital, Fuzhou, Fujian 350001, China; ^2^ Provincial Clinical College, Fujian Medical University, Fuzhou, Fujian 350001, China; ^3^ Department of Ophthalmology, Johns Hopkins School of Medicine, Baltimore, MD 21205, USA; ^4^ Department of Hepatobiliary Surgery, Fujian Provincial Hospital, Fuzhou, Fujian 350001, China; ^5^ Department of Pathology, Fujian Provincial Hospital, Fuzhou, Fujian 350001, China; ^6^ The Sidney Kimmel Comprehensive Cancer Center, Johns Hopkins School of Medicine, Baltimore, MD 21205, USA

**Keywords:** hepatocellular carcinoma, poorly differentiated, transcriptome sequencing, differentially expressed genes, carcinogenesis

## Abstract

Poorly differentiated (PD) hepatocellular carcinoma (HCC) has a worse prognosis compared to moderately differentiated (MD) and well differentiated (WD) HCC. We aimed to identify differentially expressed genes (DEGs) to explore the mechanism of PD HCC. Transcriptome sequencing was performed on tumor and adjacent non-tumorous tissues of PD, MD and WD HCC patients (3 for each group). DEGs were thus identified and functionally analyzed. Further RT-PCR was performed to validate DEGs specific for PD HCC in 47 pairs of samples (15 for PD, 18 for MD, 14 for WD). A total of 681 PD DEGs were detected, including 368 up-regulated and 313 down-regulated genes. Less DEGs were found for MD and especially for WD HCC. Through bioinformatics analysis, PD HCC DEGs were enriched in liver tissue and liver cancer cells, and in biological process and pathway including metabolism, cell cycle, translation and blood coagulation. Potential drugs and genetic perturbations were found to reverse the cancer condition. The RT-PCR results showed consistency with RNA-seq in the validation of 4 DEGs specific for PD HCC. This study detected and validated DEGs of PD HCC, which provides useful information on molecular mechanism of PD HCC for development of new biomarkers, therapeutic targets and drugs.

## INTRODUCTION

As one of the most common malignancies worldwide, hepatocellular carcinoma (HCC) represents the second-leading cause of cancer deaths globally with 745,000 deaths per year [1]. The HCC were categorized into poorly-, moderately-, and well- differentiated types. Hepatic resection is currently the most optimal choice for HCC treatment. However, poorly differentiated HCC has a worse prognosis with high recurrence rate compared to other two types [2, 3]. In addition, it is hard to discriminate poorly differentiated HCC from other two types of HCC before treatment. The lack of good diagnostic markers and therapeutic targets has rendered HCC a major challenge.

Recently, high-throughput technologies like RNA sequencing make it possible and easy to illustrate the transcriptome characteristics of cancers including HCC [4]. Some potential biomarkers for HCC were identified using transcriptome sequencing e.g. SERPINA11 whose expression is correlated with pathology stages lack documented expression profiles in liver cancer [5]. Some signaling pathways like cell cycle were also involved via analysis of transcriptome sequencing data [6]. Those studies show a great promise of exploring the molecular basis of HCC.

In this study, we performed transcriptome sequencing for patients with poorly differentiated (PD), moderately differentiated (MD) and well differentiated (WD) HCC. 3 patients diagnosed as HCC of each grades were recruited, respectively. Differentially expressed genes (DEGs) were identified through analysis of transcriptome sequencing data. DEGs were then subjected to gene set enrichment analysis. Furthermore, RT-PCR was performed to validate potential biomarkers in 15 pairs of poorly differentiated tumor and adjacent non-tumorous samples. Our investigation may provide new clues on the molecular event responsible for the progression of HCC and potential biomarkers and therapeutic targets for diagnosis and treatment of HCC patients.

## RESULTS

### Overview of transcriptome sequencing statistics

**Figure 1:**
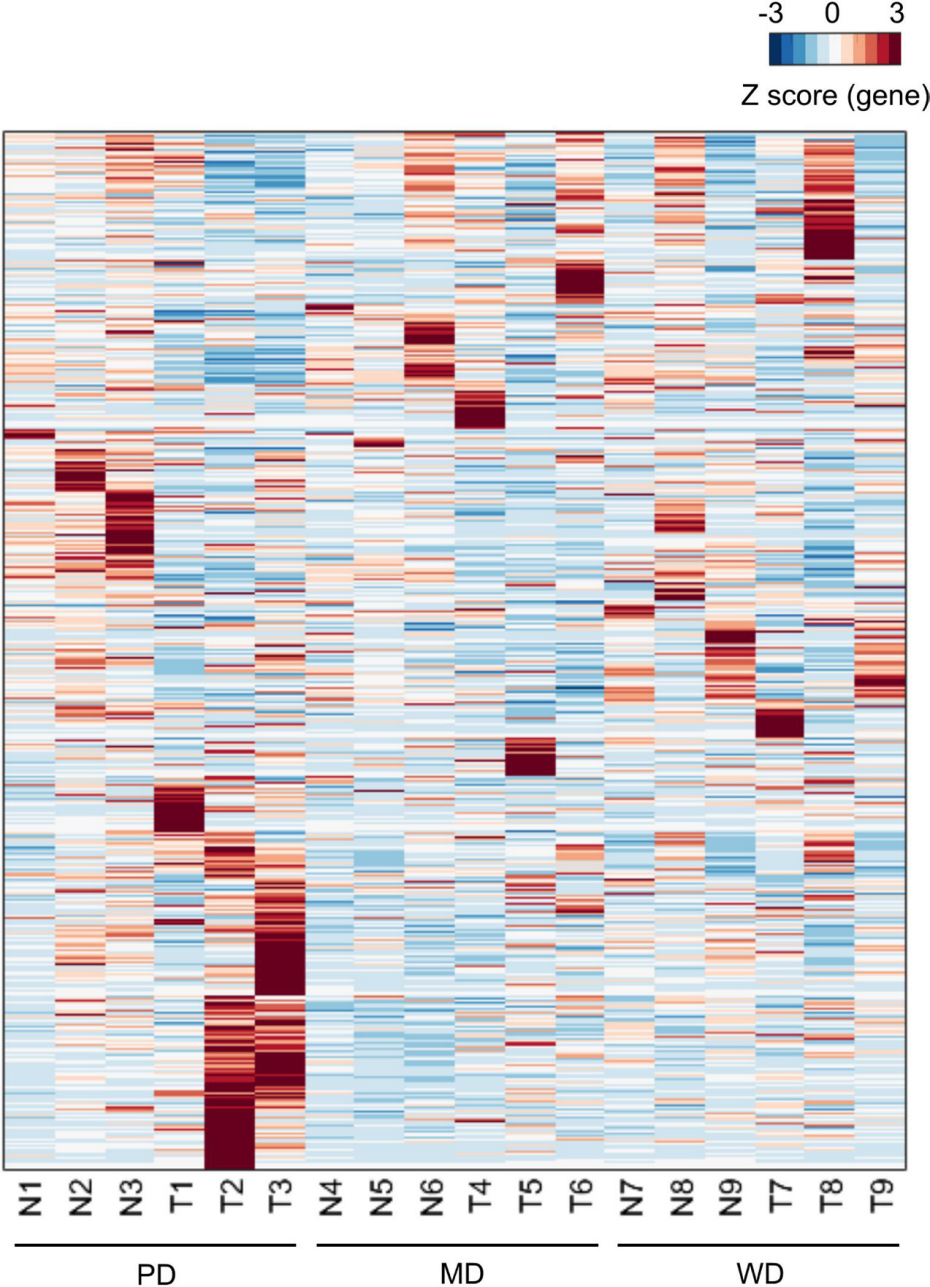
Heatmap of expressed genes For the sample labels, N and T represented matched adjacent non-tumor tissue specimens and tumor tissue specimens respectively. Each row represented of Z scores of genes across different samples.

Pair-end second-generation transcriptome sequencing was performed in 9 HCC patients. Sample characteristics were shown in Table 1. An average of 35,772,695 pair-end 125 bp clean reads was generated (Table 2). The average mapping rate was 93.17%, resulting an average coverage of depth of 32 × (Table 2). Expression levels of more than 25,200 genes were calculated using Tophat/Cufflinks (Supplementary Table 1). The heatmap of all expressed genes were drawn in Figure 1, which showed a big group of differentially expressed genes (DEGs) between tumor and adjacent non-tumorous samples of poorly differentiated HCC.

**Table 1:** Detailed characteristics of the patients

Patient	Age	Gender	Hepatitis	Serum AFP level (ng/mL)	Metastasis	Glisson capsule invasion	Tumor size (mm)	Differentiation Grade	Multiple liver nodules
***P1***	***59***	***M***	***HBV***	***670.1***	***No***	−	***35***	***Poorly***	-
***P2***	***50***	***M***	***HBV***	***12483***	***No***	+	***50***	***Poorly***	-
***P3***	***37***	***M***	***HBV***	***4.47***	***No***	+	***41***	***Poorly***	-
***M1***	***59***	***M***	***HBV***	***3.73***	***No***	−	***6***	***moderately***	-
***M2***	***69***	***M***	***HBV***	***6.9***	***No***	+	***160***	***moderately***	***synchronous***
***M3***	***61***	***M***	***NBNC***	***1.11***	***No***	+	***80***	***moderately***	-
***W1***	***62***	***M***	***HBV***	***266.5***	***No***	+	***32***	***Well***	-
***W2***	***76***	***M***	***HCV***	***5.84***	***No***	+	***70***	***Well***	-
***W3***	***19***	***F***	***NBNC***	***2.85***	***No***	−	***29***	***Well***	-
P4	48	M	NBNC	55.1	Yes	+	180	Poorly	-
P5	41	M	HBV	4.7	Yes	+	50	Poorly	-
P6	64	M	HBV	244.6	Yes	+	65	Poorly	synchronous
P7	50	M	HBV	3.03	Yes	+	55	Poorly	synchronous
P8	47	M	HBV	62.47	Yes	+	105	Poorly	-
P9	64	M	HBV	36541	Yes	+	140	Poorly	-
P10	52	M	HBV	2100	Yes	−	35	Poorly	-
P11	52	M	NBNC	5.3	No	+	65	Poorly	-
P12	37	M	HBV	1810	No	−	55	Poorly	-
P13	48	M	HBV	2.07	No	−	35	Poorly	-
P14	52	F	NBNC	2.45	No	+	22	Poorly	-
P15	58	M	HBV	6.52	Yes	+	58	Poorly	synchronous
M4	63	M	HBV	2975	No	+	70	moderately	synchronous
M5	47	M	HBV	5375	Yes	+	130	moderately	-
M6	72	M	HBV	303.8	No	+	40	moderately	synchronous
M7	66	M	HBV	60500	No	+	50	moderately	-
M8	59	M	NBNC	54.84	No	+	26	moderately	-
M9	67	M	HBV	321.1	No	+	55	moderately	synchronous
M10	71	M	HBV	4606	Yes	+	12	moderately	-
M11	18	F	HBV	37979	No	+	70	moderately	-
M12	45	M	HBV	6528	No	+	60	moderately	synchronous
M13	61	M	HBV	1.9	No	+	30	moderately	-
M14	52	M	HBV	26.53	No	+	20	moderately	-
M15	58	F	HBV	2.55	Yes	+	55	moderately	synchronous
M16	42	M	HBV	3.38	No	+	20	moderately	synchronous
M17	64	M	HBV	774.2	No	+	10	moderately	-
M18	54	F	NBNC	23784	No	+	22	moderately	-
W4	72	M	HBV	11.10	No	+	20	Well	-
W5	55	M	HBV	1.82	No	+	34	Well	-
W6	76	M	NBNC	2.1	No	+	34	Well	-
W7	67	M	NBNC	3.30	No	−	18	Well	-
W8	50	M	HBV	260.9	No	−	25	Well	-
W9	71	M	HBV	11.65	No	+	35	Well	-
W10	74	M	NBNC	5.9	No	+	30	Well	-
W11	39	M	HBV	8.10	Yes	+	22	Well	synchronous
W12	63	M	HBV	208	No	+	40	Well	-
W13	48	M	NBNC	3.07	No	−	33	Well	-
W14	66	M	HBV	82.81	No	+	34	Well	-

Note: The nine samples used for sequencing are labeled in bold and italic.

**Table 2:** Summary statistics of the transcriptome sequencing

Patient	Differentiation grade	Sample Type	Total reads	Mapped reads	Total base (bp)	Mapped base (bp)	Mappping ratio	Coverage (X)
P1	Poorly	T	35,930,408	33,819,287	4,491,301,000	4,227,410,875	94.12%	33
		N	34,902,228	32,863,973	4,362,778,500	4,107,996,625	94.16%	32
P2	Poorly	T	35,315,378	33,125,921	4,414,422,250	4,140,740,125	93.80%	32
		N	34,383,802	32,279,443	4,297,975,250	4,034,930,375	93.88%	31
P3	Poorly	T	35,532,658	33,258,212	4,441,582,250	4,157,276,500	93.60%	32
		N	34,537,914	32,152,860	4,317,239,250	4,019,107,500	93.09%	31
M1	Moderately	T	34,566,526	32,670,216	4,320,815,750	4,083,777,000	94.51%	31
		N	35,384,486	33,407,169	4,423,060,750	4,175,896,125	94.41%	32
M2	Moderately	T	34,564,642	32,385,294	4,320,580,250	4,048,161,750	93.69%	31
		N	34,771,048	32,792,475	4,346,381,000	4,099,059,375	94.31%	32
M3	Moderately	T	35,371,920	32,571,695	4,421,490,000	4,071,461,875	92.08%	31
		N	35,959,532	32,990,753	4,494,941,500	4,123,844,125	91.74%	32
W1	Well	T	34,762,386	32,701,011	4,345,298,250	4,087,626,375	94.07%	31
		N	35,314,918	33,021,170	4,414,364,750	4,127,646,250	93.50%	32
W2	Well	T	35,610,444	32,509,118	4,451,305,500	4,063,639,750	91.29%	31
		N	34,637,398	31,280,461	4,329,674,750	3,910,057,625	90.31%	30
W3	Well	T	42,279,434	39,097,967	5,284,929,250	4,887,245,875	92.50%	38
		N	40,083,384	36,852,292	5,010,423,000	4,606,536,500	91.90%	35
Average			35,772,695	33,321,073	4,471,586,847	4,165,134,146	93.17%	32

Note: T and N represent tumor and adjacent non-tumor tissue, respectively.

### Identification of DEGs between tumor and adjacent non-tumor tissues

**Figure 2:**
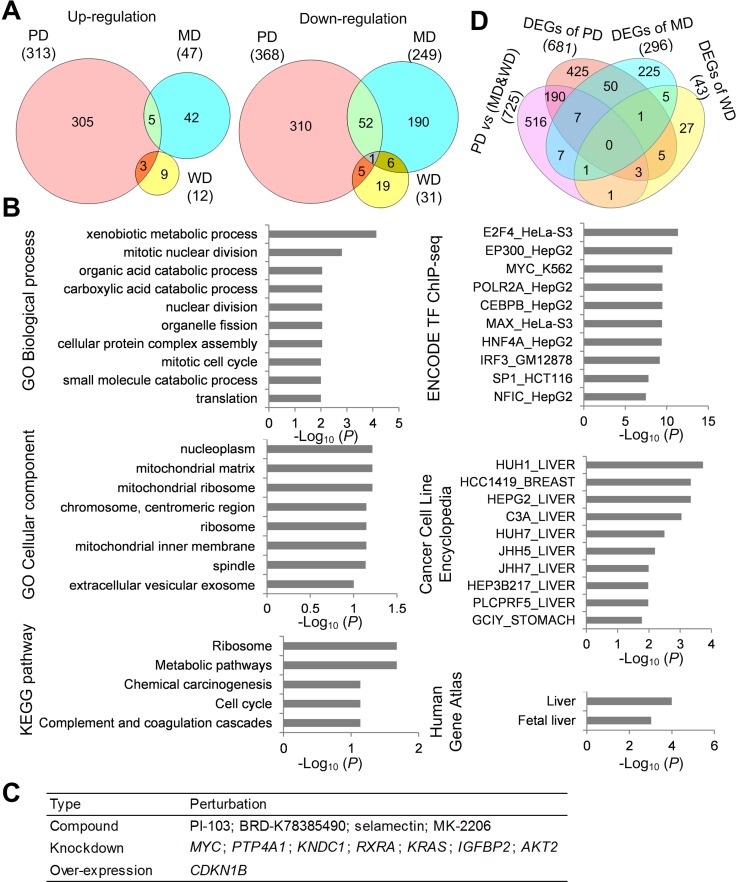
Functional characterization of DEGs (**A**) Venn diagram of DEGs between poorly differentiated (PD), moderately differentiated (MD) and well differentiated (WD) HCC. (**B**) Functional enrichment analysis of DEGs of poorly differentiated HCC. (**C**) Top 12 perturbations with high negative connectivity scores with DEGs of poorly differentiated HCC. (**D**) Venn diagram of DEGs between PD HCC tumor tissues and MD&WD tumor tissues, DEGs of PD HCC, DEGs of MD HCC, and DEGs of WD HCC.

We next detected DEGs between tumor and adjacent non-tumorous samples of poorly differentiated, moderately differentiated and well differentiated HCC. Through paired-*T* test analysis (*P value* < 0.05, Fold change > 2), a total of 1020 DEGs were detected including 372 up-regulated genes (313, 47 and 12 for poorly-, moderately- and well-differentiated HCC, respectively) and 648 down-regulated genes (368, 249 and 31 for poorly-, moderately- and well-differentiated HCC, respectively) (Figure 2A; Supplementary Table 2). Only 1 down-regulated gene (CD44) was overlapped in all 3 grades. Top 20 up-regulated genes and Top 20 down-regulated genes of poorly differentiated HCC were shown in Table 3 and Table 4, respectively.

**Table 3:** Top 20 up-regulated genes of poorly differentiated HCC

Entrez_ID	Gene Symbol	N1	N2	N3	T1	T2	T3	Group mean _exp_N	Group mean _exp_T	Group _log2FC	Group ttest _*p* value
10130	PDIA6	116.76	123.98	126.57	255.14	260.71	263.04	122.44	259.63	1.08	1.90E-05
578	BAK1	6.41	5.00	9.99	20.24	18.51	23.98	7.14	20.91	1.43	1.03E-04
1650	DDOST	53.21	72.67	59.72	115.80	139.67	121.44	61.87	125.64	1.01	6.57E-04
2786	GNG4	0.04	0.02	0.23	1.84	1.90	2.21	0.10	1.98	1.45	7.97E-04
84933	C8orf76	6.03	5.11	6.90	24.33	21.27	23.79	6.01	23.13	1.78	1.35E-03
22996	TTC39A	0.04	0.29	0.46	2.38	2.35	2.73	0.26	2.49	1.46	1.46E-03
5691	PSMB3	119.36	132.67	145.10	301.66	342.81	347.47	132.37	330.64	1.31	1.74E-03
54522	ANKRD16	1.87	2.67	2.10	6.20	7.28	7.16	2.21	6.88	1.29	2.04E-03
28998	MRPL13	13.72	14.12	18.44	69.51	61.37	73.26	15.43	68.05	2.07	2.62E-03
50854	C6orf48	28.97	70.17	66.97	111.29	140.99	150.56	55.37	134.28	1.26	2.64E-03
3094	HINT1	270.52	305.61	289.43	605.26	590.80	625.23	288.52	607.10	1.07	2.74E-03
4738	NEDD8	131.06	138.11	152.30	291.78	329.80	338.89	140.49	320.16	1.18	2.84E-03
57538	ALPK3	0.47	1.81	1.41	3.16	4.41	3.61	1.23	3.73	1.08	3.65E-03
65244	SPATS2	2.33	3.32	3.91	6.11	7.99	8.07	3.18	7.39	1.00	3.73E-03
8624	PSMG1	15.75	16.67	25.69	37.42	39.74	44.17	19.37	40.44	1.02	4.12E-03
8836	GGH	61.61	26.09	42.65	124.45	87.68	117.67	43.45	109.93	1.32	4.12E-03
23640	HSPBP1	7.35	11.32	9.61	19.05	25.08	24.27	9.42	22.80	1.19	4.27E-03
79075	DSCC1	0.34	0.51	1.13	4.16	4.12	4.17	0.66	4.15	1.63	4.36E-03
5591	PRKDC	5.80	5.46	7.02	17.28	15.77	16.09	6.09	16.38	1.29	4.50E-03
84701	COX4I2	0.22	1.13	0.23	3.16	4.83	3.73	0.53	3.91	1.69	4.56E-03

Note: T1, T2, T3 represent tumor tissue of P1, P2, P3 with poorly differentiated HCC, respectively; N1, N2, N3 represent adjacent non-tumor tissue of P1, P2, P3 with poorly differentiated HCC, respectively.

**Table 4:** Top 20 down-regulated genes of poorly differentiated HCC

Entrez_ID	Gene Symbol	N1	N2	N3	T1	T2	T3	Group mean _exp_N	Group mean _exp_T	Group _log2FC	Group ttest _*p* value
83854	ANGPTL6	25.01	28.16	28.36	4.67	8.61	8.62	27.18	7.30	−1.76	1.44E-04
2706	GJB2	14.85	15.50	12.98	2.92	3.18	1.25	14.44	2.45	−2.16	2.00E-04
23002	DAAM1	5.54	5.70	6.63	1.39	1.70	2.41	5.96	1.83	−1.30	2.23E-04
8671	SLC4A4	10.64	9.24	9.18	2.04	1.21	1.14	9.68	1.46	−2.12	5.10E-04
389643	NUGGC	8.92	9.30	8.32	1.61	2.46	1.52	8.85	1.86	−1.78	5.44E-04
80824	DUSP16	18.19	15.58	14.62	8.60	6.06	5.75	16.13	6.80	−1.13	5.97E-04
1756	DMD	19.00	20.67	22.49	1.15	4.00	4.22	20.72	3.12	−2.40	7.46E-04
2244	FGB	4822.00	2846.01	2754.51	2475.77	484.91	192.48	3474.17	1051.05	−1.72	8.24E-04
56907	SPIRE1	3.97	5.48	6.04	0.96	2.07	2.81	5.17	1.95	−1.06	1.29E-03
283537	SLC46A3	39.62	40.22	43.44	4.31	1.39	8.81	41.09	4.84	−2.85	1.29E-03
132671	SPATA18	2.87	2.41	2.67	0.12	0.02	0.20	2.65	0.11	−1.71	1.81E-03
1003	CDH5	14.79	13.86	23.11	2.30	1.73	9.11	17.25	4.38	−1.76	1.97E-03
4051	CYP4F3	116.69	77.42	71.59	57.44	8.09	7.12	88.57	24.21	−1.83	2.04E-03
9953	HS3ST3B1	5.67	5.24	4.49	0.63	0.47	0.18	5.13	0.43	−2.10	2.05E-03
10894	LYVE1	43.68	40.34	38.97	0.56	3.11	0.76	41.00	1.48	−4.08	2.12E-03
57188	ADAMTSL3	5.57	4.39	4.54	1.40	0.08	0.87	4.83	0.78	−1.71	2.25E-03
152926	PPM1K	3.52	3.35	4.35	0.93	0.81	1.39	3.74	1.04	−1.21	2.27E-03
8658	TNKS	6.36	5.58	6.40	2.33	2.03	2.20	6.11	2.19	−1.16	2.44E-03
116519	APOA5	208.64	134.96	119.75	88.24	9.53	14.19	154.45	37.32	−2.02	2.58E-03
90417	KNSTRN	2.17	3.28	4.17	7.96	6.80	8.81	3.21	7.85	1.07	1.94E-02

Note: T1, T2, T3 represent tumor tissue of P1, P2, P3 with poorly differentiated HCC, respectively; N1, N2, N3 represent adjacent non-tumor tissue of P1, P2, P3 with poorly differentiated HCC, respectively.

### Significantly enriched functions for DEGs of poorly differentiated HCC

DEGs of poorly differentiated HCC were subjected to functional enrichment analyses to illustrate their biological function characteristics using Enrichr tool [7]. The top enriched terms for categories were shown in Figure 2B (*P value* < 0.1). From Human Gene Atlas and Cancer Cell Line Encyclopedia, those DEGs were enriched in liver tissue and various liver cancer cells. The enriched biological process and pathway included metabolism, cell cycle, translation and blood coagulation. The DEGs tended to lie in mitochondrial, chromosome and ribosome. From the ENCODE TF ChIP-seq data, 5 transcription factors in liver cancer cell HEPG2 were enriched to regulate the poorly differentiated DEGs.

### Potential perturbations for reverse of abnormal gene expression change of poorly differentiated HCC

LINCS is a collection of signatures of gene expression for a broad range of conditions such as drug treatment, ligand treatment, gene knockdown, and gene over-expression in many different types of human cells [8]. We inputted the up-regulated genes and down-regulated genes into lincscloud to find perturbations that had opposite gene expression change with differentiated HCC. Top 12 perturbations with high negative connectivity scores (Figure 2C), which included 4 compounds, 7 knockdown genes and 1 over-expression gene.

### Comparison of tumor tissues between PD HCC and MD&WD HCC

Besides DEGs between tumor and adjacent non-tumorous samples, we compared tumor tissues between PD HCC and MD&WD HCC. As a result, 725 differentially expressed genes were identified (Supplementary Table 2); among them, 209 were identified as DEGs when comparing tumor and adjacent non-tumorous samples (Figure 2D). Gene ontology enrichment analysis was also done based on this 725-gene list. Similarly, these genes were enriched in organelle fission, mitotic nuclear division, positive regulation of cell cycle phase transition and serine family amino acid catabolic process.

### Validation of DEGs specific for poorly differentiated HCC by RT-PCR

**Figure 3:**
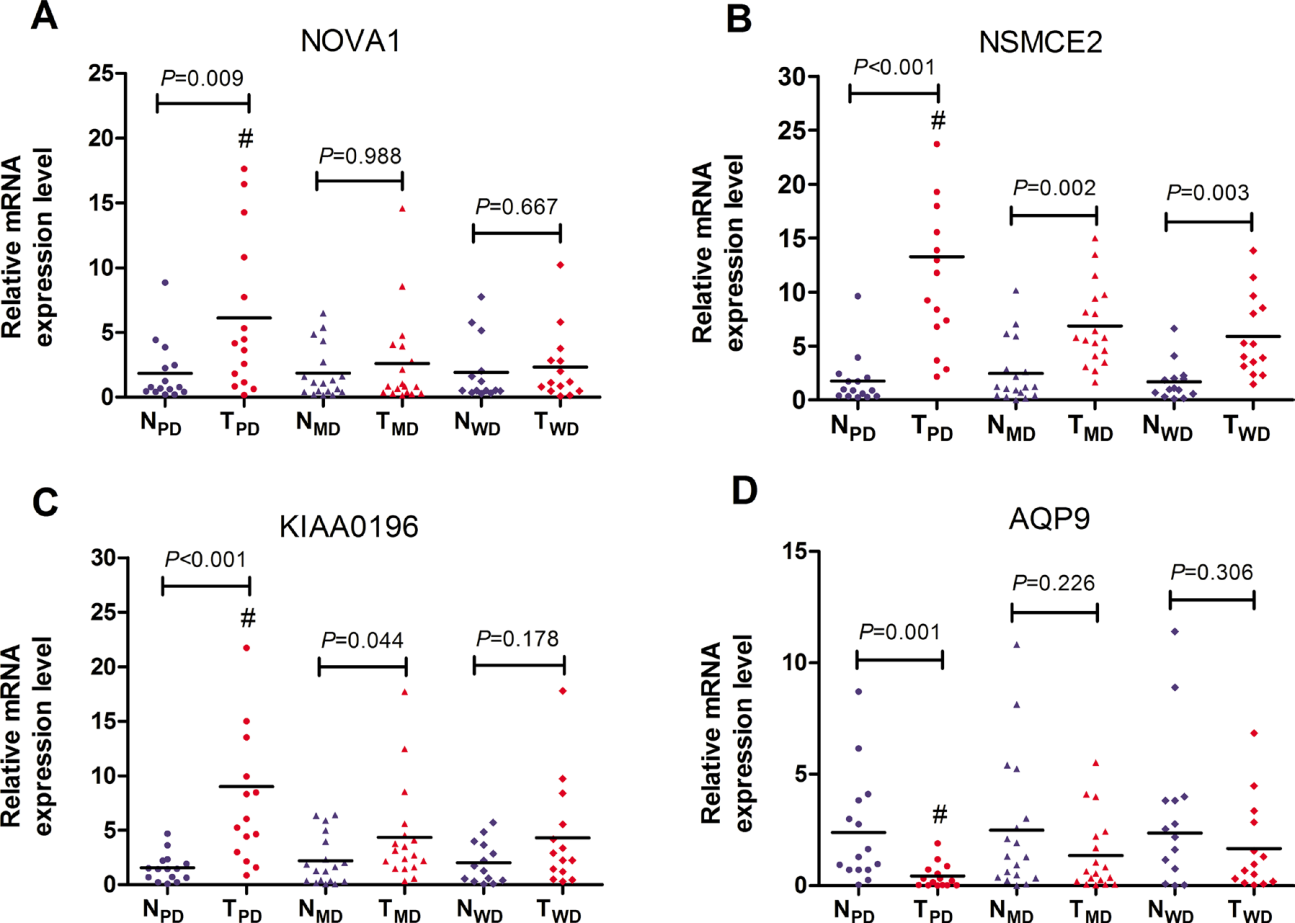
RT-PCR validation of 4 DEGs specific for poorly differentiated HCC GAPDH mRNA was used as an internal control. Expression data were obtained as 2^−ΔΔCT^ relative to adjacent non-tumor tissue values. N_PD_ and T_PD_ represented matched adjacent non-tumor tissue specimens (*n* = 15) and poorly differentiated HCC tissue specimens (*n* = 15) respectively. N_MD_ and T_MD_ represented matched adjacent non-tumor tissue specimens (*n* = 18) and moderately differentiated HCC tissue specimens (*n* = 18) respectively. N_WD_ and T_WD_ represented matched adjacent non-tumor tissue specimens (*n* = 14) and well differentiated HCC tissue specimens (n=14) respectively. (**A**) NOVA1; (**B**) NSMCE2; (**C**) KIAA0196; (**D**) AQP9. ^#^*P* < 0.05 *vs* T_MD_ and T_WD_.

4 DEGs specific for poorly differentiated HCC as well as related with cell cycle or proliferation were validated by RT-PCR. In the discovery phase, the expressions of NOVA1, NSMCE2 and KIAA0196 were significantly up-regulated, while expression of AQP9 was significantly down-regulated in poorly differentiated samples, as compared with that in adjacent non-tumorous samples, moderately differentiated samples and well differentiated samples (*P* < 0.05) (Table 5). RT-PCR results showed that all these 4 genes were successfully validated (Figure 3), and the dysregulation trend matched with those observed in the RNA-seq data.

**Table 5:** 4 DEGs specific for poorly differentiated HCC

Entrez ID	Gene Symbol	N1	N2	N3	N4	N5	N6	N7	N8	N9	T1	T2	T3	T4	T5	T6	T7	T8	T9	*t* test *p* value
4857	NOVA1	0.45	0.92	0.60	0.46	0.33	0.60	0.34	0.34	0.68	8.90	4.66	6.21	0.53	0.54	2.34	0.93	0.14	0.19	3.92E-022
286053	NSMCE2	7.21	11.26	12.07	10.67	7.07	14.89	10.64	14.13	7.46	49.75	80.27	61.78	13.90	19.52	17.62	16.57	28.10	10.60	2.74E-02
9897	KIAA0196	11.23	9.64	12.62	7.93	9.70	11.73	7.95	13.44	7.68	29.40	34.53	27.14	10.02	5.18	10.03	11.86	14.91	6.73	6.78E-03
55039	AQP9	333.45	217.87	178.65	419.54	364.99	429.47	445.80	385.51	257.32	25.06	15.89	19.74	174.82	215.45	188.60	152.81	199.10	232.73	1.32E-03

Note: N1, N2, N3 and T1, T2, T3 represent adjacent non-tumor tissue and tumor tissue of P1, P2, P3 with poorly differentiated HCC, respectively. N4, N5, N6 and T4, T5, T6 represent adjacent non-tumor tissue and tumor tissue of M1, M2, M3 with moderately differentiated HCC, respectively. N7, N8, N9 and T7, T8, T9 represent adjacent non-tumor tissue and tumor tissue of W1, W2, W3 with well differentiated HCC, respectively. *p* value refers to T1, T2, T3 vs N1, N2, N3, N4, N5, N6, N7, N8, N9, T4, T5, T6, T7, T8, T9 by *t* test.

## DISCUSSION

We have applied the transcriptome sequencing approach to illustrate the gene expression characteristics of poorly differentiated HCC. The number of DEGs for poorly differentiated HCC is significantly bigger than that of other two stages. Those DEGs are enriched in liver tissue and cells, which is easily understood. Pathway analysis showed that two pathways, cell cycle and complement and coagulation cascades, were overrepresented with DEGs. Deregulation of the cell cycle pathway is expected since uncontrolled cell division is the major character of cancer cells. As for the complement and coagulation cascades pathway, consistent with our results, both gene expression [9] and proteomics [10] analysis have shown that this pathway is related to the pathogenesis of HCC. Some transcription factors were found to regulate the DEGs. For example, HNF4-α could represent a central regulator of gene transcription in hepatocytes, and a strong candidate to be involved in liver cancer cell development [11]. In addition, we also compared tumor tissues between PD HCC and MD&WD HCC. And similarly, the differentially expressed genes are enriched in the process of cell cycle and division.

After query of the poorly differentiated HCC signature in the LINCS server in our study, some compounds, knockdown genes and overexpression genes were found to have strong negative connections with the signature. PI-103 is a potent ATP-competitive dual inhibitor of phosphatidylinositol 3-kinase (PI3K) and mTOR (mammalian target of rapamycin). The combination of PI-103 and sorafenib was found to inhibit hepatocellular carcinoma cell proliferation by blocking Ras/Raf/MAPK and PI3K/AKT/mTOR pathways [12]. MK-2206 is an oral selective allosteric inhibitor of Akt that targets all three isoforms of human Akt (Akt-1, Akt-2 and Akt-3). MK2206 was found to inhibit hepatocellular carcinoma cellular proliferation via induction of apoptosis and cell cycle arrest [13]. Among 8 genetic perturbations, 5 genes are enriched in pathways in cancer or PI3K-Akt signaling pathway (p<0.001). Myc proto-oncogene protein (MYC) as a transcription factor can activate the transcription of growth-related genes. HCC is frequently associated with overexpression of MYC. In this study, MYC is up-regulated in the poorly differentiated HCC. Knockdown of oncogenic KRAS was found to suppress tumor growth in non-small cell lung cancers [14]. As mentioned, AKT2 is the target of MK2206, knockdown and inhibition of AKT2 may both help to reverse HCC. Overexpression of cyclin dependent kinase inhibitor 1B (CDKN1B) has the reverse effect in our study. CDKN1B binds to and prevents the activation of cyclin complexes, and thus controls the cell cycle progression at G1 [15]. All those perturbations may be the potential therapies or targets for treatment of HCC. In the validation cohort, further RT-PCR was performed to validate 4 DEGs specific for poorly differentiated HCC, of which NOVA1, NSMCE2 and KIAA0196 were significantly up-regulated, while AQP9 was significantly down-regulated in poorly differentiated samples, as compared with that in 9 adjacent non-tumor samples, 3 moderately differentiated samples and 3 well differentiated samples. The RT-PCR results showed consistency with RNA-seq. Interestingly, these DEGs were closely related with cell cycle or proliferation. High expression of NOVA1 was found correlated with poor survival rate and increased recurrence rate in HCC [16]. NSMCE2 was required for G1-S transition in breast cancer cells and manipulation of NSMCE2-mediated sumoylation may alter the growth rates of breast cancer cells [17]. KIAA0196, involving in meiosis-related spindle assembly [18], has been showed increased expression in clinical prostate carcinomas and also amplified in 30-40% of xenografts and hormone-refractory tumors [19]. However, AQP9 has been found to be down-regulated in hepatocellular carcinoma and its over-expression suppresses hepatoma cell invasion through inhibiting epithelial-to-mesenchymal transition [20]. In conclusion, this study explored the molecular mechanism of hepatocarcinogenesis through assessment of RNA seq data of HCC and validation of 4 DEGs specific for poorly differentiated HCC in an independent cohort. It provides useful information on the transcriptomic landscape as well as a mechanistic overview of HCC. Our findings offer novel insights and useful support in biomarker development and suggest new potential targets in poorly differentiated HCC characterization.

## MATERIALS AND METHODS

### Ethics statement

Our study design was approved by the Ethics Committee of the Fujian Provincial Hospital. Written informed consent was obtained from all subjects.

### Subjects

47 subjects were diagnosed as primary HCC in the Fujian Provincial Hospital (Table 1), of which 33 subjects were present with cirrhosis on the non-neoplastic background, including 29 subjects with hepatitis B virus (HBV), 1 subjects with hepatitis C virus (HCV) and 3 subjects with NBNC. HBV related tumors were defined according to the presence of HB surface antigen (HBsAg) in serum, and HCV related tumors were according to the presence of antibody to HCV (HCVAb) in serum. NBNC tumor was defined according to the absence of both HBsAg and HCVAb in serum. Primary tumor and adjacent non-tumorous samples were obtained from all patients who underwent surgical tumor resection. All samples were frozen immediately at −80°C until RNA extraction. Total RNA was isolated by using RecoverAll™ Total Nucleic Acid Isolation Kit (Life Technologies, Carlsbad, CA, USA). Integrity of RNA was assessed by Agilent 2100 bioanalyzer (Agilent, Santa Clara, CA, USA). RNA from nine samples was subjected to sequencing and all samples were used in the validation experiments.

### Transcriptome sequencing

Sequencing libraries were prepared by using *TruSeq RNA Sample Prep Kit (Illumina, San Diego, CA, USA)* according to standard protocols. Briefly, total RNA was firstly randomly fragmented and poly-A-selected. Secondly, the RNA fragments were reverse transcribed to cDNA, end-repaired and ligated with adapters. The libraries then underwent size selection, PCR and purification. The quality of libraries was assessed by using Bioanalyzer 2100 (Agilent, Santa Clara, CA, USA). Sequencing was then performed on an Illumina HiSeq2500 sequencer with 125 bp pair-end reads.

### Reads processing

Raw sequencing reads were firstly filtered for adapters and ribosomal RNA. Reads containing five or more low quality (quality score < 20) bases were also removed. The remained high-quality reads were then aligned to human genome (hg19) by using Tophat [21]. The mapped reads were then subjected to alignment against the human transcriptome (Ensembl, GRCh37.73). Gene expression level measured by FPKM (fragments per kilobase per million) was calculated by Cufflinks [22].

### Differentially expressed genes (DEGs) analysis

For each differentiated HCC group, DEGs between the tumor and matched non-tumorous tissues were identified with pair-wise *t* test and the significant threshold was set as adjusted *p-value* of less than 0.05 and |log_2_(fold change, FC)| > = 1. Enrichr was used to do functional enrichment analysis like Gene Ontology (GO) and KEGG pathway [7]. The significant threshold for enrichment was set as *p* < 0.1. In addition, the list of up-regulated genes and down-regulated was uploaded into lincscloud [8] to find perturbations which can reverse the cancer condition. Using similar methods (*t* test instead of paired *t* test), DEGs were identified and analyzed between tumor tissues of PD HCC and those of MD&WD HCC.

### DEGs specific for poorly differentiated HCC validated by RT-PCR

DEGs specific for poorly differentiated HCC (Compared with 3 matched adjacent non-tumor tissue of poorly differentiated HCC, P<0.05, |log_2_(fold change, FC)| > = 1; Compared with all adjacent non-tumor tissue, 3 moderately differentiated tissue and 3 well differentiated tissue, P<0.05, |log_2_(fold change, FC)| > = 1) were subjected to validation using RT-PCR. The validation cohort included 15 poorly differentiated HCC, 18 moderately differentiated HCC and 14 well differentiated HCC (Table 1). For the RT-PCR reactions, total RNA was converted to cDNA with random hexamer primers using the High-Capacity cDNA Reverse Transcription kit (Applied Biosystems, Foster City, CA, USA). Real-time PCR was performed with SYBR Green I (Applied Biosystems, United States) on ABI 7300 (Applied Biosystems). The primers used were described in Table 6.

**Table 6:** Primers used in this study

Gene	Primers (5′–3′)	Length (bp)
NOVA1	GACCAATACGGGCGAAGACG	295
	CTGGGGTTGTAGAATGCTGACTG	
NSMCE2	AGACCAACTTCACCTGCCCC	131
	CTTTTTCTTCCGCTTTTGCCTG	
KIAA0196	GAGGGAGGGGTGGAAACTGG	208
	ATTGTGAGGCGGACCGACTAC	
AQP9	TGGAGGGGTCATCACTATCAAT	226
	CATAAGTCCATCATAGTAAATGCCAAA	

### Statistical analysis

*T-test* was used to compare continuous measurement data with a normal distribution between two groups. Mann-Whitney *U* test was used to evaluate continuous data with a non-normal distribution. Statistical analyses were performed with the SPSS13.0 software (SPSS, United States). *P* < 0.05 was considered statistically significant.

## SUPPLEMENTARY TABLES







## Abbreviations

HCC: hepatocellular carcinoma
PD: poorly differentiated
MD: moderately differentiated
WD: well differentiated
DEGs: differentially expressed genes
GO: Gene Ontology
*KEGG*: Kyoto Encyclopedia of Genes and Genomes
PI3K: phosphatidylinositol 3-kinase
Akt: protein kinase B
mTOR: mammalian target of rapamycin
MAPK: mitogen activated kinase-like protein
MYC: Myc proto-oncogene protein
CDKN1B: cyclin dependent kinase inhibitor 1B
FPKM: fragments per kilobase per million
HBV: Hepatitis B virus
HBsAg: HB surface antigen
HCV: Hepatitis C virus
NBNC tumor: non-B non-C tumor
NOVA1: NOVA alternative splicing regulator 1
NSMCE2: NSE2/MMS21 homolog, SMC5-SMC6 complex SUMO ligase
AQP9: aquaporin 9
