# Supplementary material for: Identification and functional analysis of differentially expressed genes in poorly differentiated hepatocellular carcinoma using RNA-seq
Source: Oncotarget. 2017 Mar 21;8(22):35973–83. doi: 10.18632/oncotarget.16415 (PMC5482631; doi:10.18632/oncotarget.16415)
Supplement: Supplementary file 1 [file oncotarget-08-35973-s001.pdf]

Identification and functional analysis of differentially expressed genes in poorly differentiated hepatocellular carcinoma using RNA-seq

Supplementary Materials

Supplementary Table 1: The RPKM value of each gene for 18 paired samples. See Supplementary_Table_1

Supplementary Table 2: Differentially expressed gene list. See Supplementary_Table_2
